# Assessing the utility of autofluorescence-based pulmonary optical endomicroscopy to predict the malignant potential of solitary pulmonary nodules in humans

**DOI:** 10.1038/srep31372

**Published:** 2016-08-23

**Authors:** Sohan Seth, Ahsan R. Akram, Paul McCool, Jody Westerfeld, David Wilson, Stephen McLaughlin, Kevin Dhaliwal, Christopher K. I. Williams

**Affiliations:** 1 University of Edinburgh, School of Informatics, Edinburgh, EH8 9AB, UK; 2 University of Edinburgh, Queens Medical Research Institute, MRC Center for Inflammation Research, Pulmonary Molecular Imaging Group, Edinburgh, EH14 4TJ, UK; 3 Heriot-Watt University, School of Engineering and Physical Sciences, Edinburgh, EH14 4AS, UK; 4 Community Health Network, Community South Hospital, Indianapolis, IN 46227, USA

## Abstract

Solitary pulmonary nodules are common, often incidental findings on chest CT scans. The investigation of pulmonary nodules is time-consuming and often leads to protracted follow-up with ongoing radiological surveillance, however, clinical calculators that assess the risk of the nodule being malignant exist to help in the stratification of patients. Furthermore recent advances in interventional pulmonology include the ability to both navigate to nodules and also to perform autofluorescence endomicroscopy. In this study we assessed the efficacy of incorporating additional information from label-free fibre-based optical endomicrosopy of the nodule on assessing risk of malignancy. Using image analysis and machine learning approaches, we find that this information does not yield any gain in predictive performance in a cohort of patients. Further advances with pulmonary endomicroscopy will require the addition of molecular tracers to improve information from this procedure.

A pulmonary nodule is defined as a focal rounded or irregular opacity in the lung, which can be well or poorly defined, measures less than 30 mm, is surrounded by aerated lung and is not associated with atelectasis or lymph node enlargement (see Fig. 1)^1^. They are common findings on computed tomography (CT) scans and cause both clinical and diagnostic uncertainty as they may represent benign disease or an early treatable lung cancer. Lung cancer remains the most common cancer in men worldwide and the fourth most common cancer in women in terms of incidence, and the most common cause of cancer-related deaths in men and second to breast cancer in women^2^. If a pulmonary nodule is diagnosed as malignant then treatment at early stage (such as stage I) offers a 73% chance of 5-year survival, whereas in late stage disease (such as stage IV) this is reduced to 13%^3^. Consequently there has been considerable interest in the early identification of patients with lung cancer. However, no single clinical variable or sign seen on radiological assessment can inform us whether a nodule is benign or malignant with absolute certainty, and current recommendations rely on the assessment of risk using a combination of clinical and radiological variables^4^,^5^ (see supplementary information on *risk calculators* for more details). These risk calculators demonstrate good operator characteristics in clinical cohorts^4^,^5^, but for individual patients the risk ascribed may still require progression to unnecessary invasive tests for benign diagnosis. With the increased use of CT scans in clinical practice^6^, and the targeted screening of high risk individuals for lung cancer,^7^ the detection of nodules will increase, and observational management may extend to four years of observation^8^ causing significant uncertainty over a long period of time. Therefore, any minimally invasive method of obtaining a more definitive diagnosis has the potential to minimise many years of CT surveillance, prevent (where unnecessary) or expedite (where necessary) surgical treatments, and thus reduce harm in both groups.

Bronchoscopy with techniques including endobronchial ultrasound and navigational technology has meant almost all of the lung parenchyma can be accessed by endobronchial means. Therefore, new technologies that allow confocal imaging of the lung parenchyma at cellular resolution by the passage of a thin fiber down the working channel of a bronchoscope, such as fibered confocal fluorescence microscopy (FCFM)—an optical endomicroscopy technique—may have a potential role in the management of patients with nodules (see supplementary information on *FCFM imaging* for more details), but this role has not been fully defined. Pulmonary FCFM imaging has been performed on patients with respiratory disease in multiple centers around the world and has an excellent safety profile^9^. Furthermore, in pulmonary FCFM imaging, some groups have reported the use of computer-aided analysis for the classification of images from healthy individuals versus pathological conditions^10^,^11^,^12^ (see supplementary information on *FCFM image analysis* for more details). These studies have found that the healthy alveolar structure can be discriminated from pathological quite effectively in *label-free* (autofluorescence only) FCFM imaging (86.3% and 95.1% for never smokers and current or ex-smokers respectively^12^). Further work by the same authors^10^,^11^ on separating normal lungs (nonsmoking or smoking healthy) from pathological lungs (diagnosed with lung disease from abnormal growth detected via CT scan) from FCFM images has demonstrated that classification between pathological and healthy in both current or ex-smokers and never smokers can be improved further using advanced image analysis and machine learning tools. Finally, it has also been demonstrated that with the application of methylene blue (a contrast agent instead of label-free) and using the Cellvizio system operating at 660 nm wavelength, the diagnosis of bronchial cancerous lesions can have a 90% classification accuracy^13^. However, there have been no reports of *label-free* (autofluorescence only) FCFM to determine benign from malignant nodules in an automated manner. Therefore, the aim of this study was to assess the efficacy of automated computational analysis of FCFM images from a clinical cohort of patients with pulmonary nodules in improving the operating characteristics of the available nodule risk calculators.

## Methods

### Dataset: demographic, clinical, radiological and imaging information

All data described includes retrospective analysis of a prospectively collected cohort. The study was approved by the Western Institutional Review Board (Puyallip, WA, USA). All procedures were undertaken using standard bronchoscopy, with the aid of superDimension^TM^Navigation System (Covidien Inc., MN, USA) and imaging with 488 nm Cellvizio^TM^(Mauna Kea Technologies, Paris, France) system. We display each variable name as variable name. For each patient, clinical data, including patient demographics, such as age and sex, and clinical risk factors for malignancy, such as smoking history (smoker and smoking pack years), previous extracthoratic cancer, and family history of lung cancer, were recorded in a blinded fashion. Furthermore, CT scans of the index presentation were independently reviewed on a picture archiving and communication system (PACS) (Carestream Vue PACS, 11.4, Rochester NY, USA) and the maximal lateral diameter (nodule size) of nodules on axial scanning were recorded, as well as the number of nodules, location of nodule (upper lobe), margin of nodule (spiculation), density of nodule (nodule type) and presence of emphysema. Malignant lesions were confirmed by either one of i) biopsy, ii) brushing of area or iii) washings of area confirming the presence of malignant cells, or the growth of a nodule during an interval scan and subsequent confirmation of malignancy. A nodule was considered benign if there was either i) no evidence of malignancy on histology/cytology and there was no interval growth (or resolution) on CT follow up for up to two years or ii) the pathology confirmed an alternative non-malignant diagnosis. All pathology results were provided by the pathology department of the Columbus Lung Institute, Indiana, USA. The nodule cohort consisted of 112 patients: of these 12 were excluded as the FCFM videos were corrupted and 9 were excluded as the nodule was not reached on FCFM imaging. Therefore the final cohort consisted of 91 complete patient datasets, of which 25 (27%) were due to malignant cause, and 66 (73%) due to a benign cause. For each video, *on-target frames* (where the distal end of the fibre had reached the nodule) were extracted manually to remove non-relevant information of adjacent normal lung, bronchial imaging or movement artefact. On-target frames were not necessarily contiguous, i.e., the operator could visit the nodule multiple times, and we extracted all such segments, i.e., each video can have multiple on-target sequences. In total, across the 91 FCFM videos a total number of 16795 frames (quartiles number of frames per video: 97, 159 and 219) were considered on target (see supplementary information on *representative frames* for examples of on-target frames). FCFM frames are circular with the dimension of the enclosing square being about 500 × 500 pixels.

### Feature extraction

From a review of the existing literature we utilize three feature extraction strategies: local binary patterns (LBP), scale-invariant feature transformation (SIFT), and scattering transformation. For each video, we extract image features from each frame independently, and then combine them (as an average over the feature space) to represent the video as a whole. Thus, the feature extraction does not explicitly depend on the number of frames per video. Additionally, we observe that the distribution of the number of frames per video for benign and malignant subjects are similar (t-test p-value 0.26).

### Local binary patterns

Local Binary Patterns (LBP) are intensity scale and rotation invariant (but not spatial scale invariant) features extensively used in texture classification^14^. LBP finds local patterns around each pixel of an image and encodes them as a binary vector. To elaborate, the intensity values at equispaced angles 2*πp*/*P* where *P* ∈ {1, …, *P*} on a circle of radius *R* around a pixel is compared with the intensity value at the center pixel. For each of these values, if it is greater than or equal to the center value then it is represented as 1 and 0 otherwise. This results in a *P* dimensional binary vector for each pixel in the image (for which a circle can be drawn). To make this representation rotation-invariant the binary vectors that are invariant to circular bit shift are combined together. Additionally, it is observed that there are only a very few binary vectors that appear commonly in the images, and the rest are usually less informative^14^. These dominant vectors are called ‘uniform’ and are identified to have less than 3 bit transitions, e.g., 00110000 has two bit transitions. Ojala *et al*.^14^ show that better classification is attained when only the uniform vectors are considered and the rest are accumulated as a miscellaneous vector. These binary vectors are represented as integers, and the image is represented as a histogram over these integers. It is also suggested to perform a multiresolution analysis where features are extracted over multiple radii (since the features are not spatial scale invariant) with *P* varied accordingly, and concatenating the resulting histograms. Since our frames are circular rather than square, we modify the original implementation slightly. We extract binary vectors at each pixel within the circle. A more cautious implementation would ignore pixels at and around the boundary given the radius of the circle, but we ignore this for simplicity since such pixels are about 1% of total pixels. For each video, we take the mean of histograms over all on-target frames, which is also a histogram (by virtue of the mean). We extract LBP histograms with following (*R*, *P*) combinations (1, 8), (2, 16), (3, 24), and (4, 24) and concatenate them to get a 80 dimensional feature vector.

### Scale-invariant feature transformation

The scale-invariant feature transformation (SIFT) is another widely popular feature extraction tool applied in object recognition^15^. The core idea of SIFT is to find a set of *scale and rotation invariant* features that are representative of the image. Each SIFT feature is a 128 dimensional vector (4 × 4 histograms over 8 quantized angles) that reflects the gradients of the image around a *keypoint* along the quantized angles, and each image is represented as a collection of SIFT features extracted at appropriate keypoints. This process is repeated over all training images. The resulting SIFT features from all training images are clustered, and each cluster center is recognized as a *visual word*. Finally, both the training and testing images are represented as histograms over the visual words by assigning their respective SIFT features to visual words. The number of clusters is user-defined. Dense SIFT^16^ is a modification of SIFT where the SIFT features are extracted at equispaced pixels of the image (instead of selected keypoints) and at fixed scale and rotation (0 radians) to simplify computation. This is repeated over multiple scales to induce scale invariance, and the SIFT features (over all scales) are pooled together before clustering. Since we have multiple videos, each with multiple frames, extracting SIFT features for multiple scales at each pixel location proves to be quite memory intensive. Instead, for each video, we extract SIFT features at random locations (within the circle), at random scales (from {2, 4, 6, 8, 10}) and at random on-target frames. We limit the maximum number of SIFT features per video to 8192. Given a set of training videos, we find 1024 visual words. Thus, we represent each training and testing video as a histogram over a 1024 dimensional vector. We also explored different number of clusters (512 and 2048) and found them to perform worse.

### Scattering transformation

The scattering transformation is a relatively novel feature extraction method that resembles a convolutional neural network with known filters which are dilated and rotated wavelets of a given family, i.e., 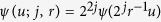, where *j* is the magnitude of dilation, *r* is the rotation, and *ψ* is the mother wavelet, e.g., Morlet wavelet^17^. At each layer of the network the scattering transformation consists of two steps, first, transforming the output image of the previous layer (with a given dilation and rotation) with the series of filters with increased dilation (relative to the previous layer), i.e., 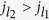 if *l*_2_ > *l*_1_ where *l* denotes layer, and all possible rotations (usually *L* equispaced ones between 0 and *π*), and second, taking the modulus of the resulting image. This resulting image is then passed on to the next layer, and a smoothed (with a filter 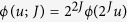) and downsampled (by 2^*J*^ where *J* is the total number of dilations) image (i.e., a set of coefficients) is kept as a feature. Thus the total number of features extracted by scattering transformation with *J* dilations, *L* rotations, and *m* layers is 
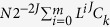
 where *N* is the number of pixels in the image. The scattering transformation parameters can be chosen by cross-validation, but some default values are often used, e.g., 2*J* = log_2_*N* such that at each layer the smoothing and downsampling only results in a single coefficient. Following existing literature^13^, we extract the largest square within each imaging circular image, and resize it to 128 × 128, and choose *J* accordingly. We use *m* = 2 and *L* = 8 which results in 1401 coefficients. For each video we take the mean of scattering features over on-target frames. Since different videos have different dynamic ranges, we normalized each frame to [0, 1] before extracting scattering features. We also experimented with transforming the image with *histogram equalization* before extracting the scattering features, however, this did not improve the performance.

### Classification

From a review of existing literature we use the following three classifiers: Lasso generalized linear model (GLM), Gaussian process classifier (GPC), and random forest (RF). For all three classifiers, we use the standard Matlab implementations. Discriminative classifiers, such as the three above, can implicitly handle non-extreme class imbalance (by extreme class imbalance we imply worse than 10:1^18^). Since the class proportion in our cohort of benign and malignant subjects is rather modest (3:1), explicit correction has not been applied. Also, in the context of feature extraction, LBP and scattering features are extracted for each frame separately, and therefore, they do not depend on the class proportions. SIFT features, on the other hand, are extracted from the entire training set, and in case of extreme class imbalance they can be extracted for each class separately.

### Lasso Generalized Linear Model

Least absolute shrinkage and selection operator (lasso) generalized linear model (GLM) solves the following problem,


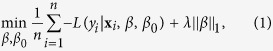


where *n* is the number of samples, (*β*_0_, *β*) is the set of coefficients to be learned, *λ* is the regularization parameter which penalizes non-sparse solutions, and *L* is the log-likelihood of a suitable probabilistic model^19^. We use log-likelihood of the Bernoulli distribution as *L*, and the logit *link* function to relate the output of linear function 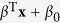 to a valid probability value. We use 5-fold cross validation to choose the best value of *λ* among 100 potential candidates between 0 and *λ*_max_ (the smallest *λ* that gives *β* = 0). We use Lasso GLM rather than standard logistic regression to tackle the *large p small n problem*, i.e., a large feature set and small sample size. We use the implementation lassoglm in Matlab.

### Gaussian process classifier

Gaussian process classifier (GPC) is a nonlinear classification strategy that assumes the following model,


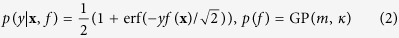


where GP(*m*, *κ*) denotes a Gaussian process with mean 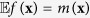 and covariance function 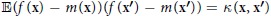
20. We use a constant mean function *m*(**x**) = *m*, and an isotropic squared exponential (Gaussian) kernel (diagonal covariance *σ***I**). We learn the hyperparameter values (mean *m* and width *σ* and height *s* of the squared exponential kernel) by maximizing the marginal likelihood function. We use the mean output value, median inter-sample distance, and 1 as initial guesses for the hyperparamers. We use expectation propagation to approximate the posterior Gaussian process. We use the Matlab implementation available at http://www.gaussianprocess.org/gpml/code/matlab/doc/.

### Random forest

Random forest (RF) is an *ensemble* of *decision trees*^21^. Each decision tree is formed by creating a split using the best predictive variable selected from a random subset of variables untill the leaf node has too few samples to split. We set the minimum samples at the leaf node to 3, and at each node sample ceil 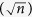 variables randomly from the original pool of variables. We use the implementation TreeBagger in Matlab with 100 trees.

### Combining classifiers

We have two information sources for each subject: the clinical and radiological information comprising 12 features, and the FCFM video information where the number of features depends on the feature extraction strategy utilized. Our goal is to combine these two information sources, and observe if we do better. This can be done in two ways, first, concatenating the information sources since both are vectors and then using a classifier; or second, combining the outputs of two classifiers learned from the two information sources separately. We chose the latter since the length of the vectors from two information sources vary significantly, e.g., 12 versus of the order of 100–1000, and finding relevant variables for prediction becomes challenging given only a few training samples, e.g., of the order of 100 (see Results section).

Let *y*_0_ and *y*_1_ be the indicators for benign and malignant classes respectively, and let **x**_1_ and **x**_2_ be the input vectors for clinical and imaging features respectively. We combine the probability of malignancy *p*(*y*_1_|**x**_1_) given by either risk calculator^4^,^5^, and probability of malignancy *p*(*y*_1_|**x**_2_) given by any one of the imaging based classifier in one of the following ways,^22^,^23^


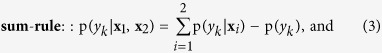



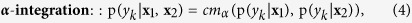


where *c* is a normalizing constant, P(*y*_*k*_)’s are the prior class probabilities, and *m*_*α*_ is function of two probability values that includes many standard combinations such as arithmetic mean (*α* = −1), geometric mean (*α* = 1), min and max (*α* = ∞, −∞) etc. as special cases (see supplementary information on *combining classifiers* for more details).

We do not need to explicitly train a classifier on the clinical and radiological information source since we have access to well-established risk calculators. We utilize cross-validation to test the performance of imaging information. To elaborate, we divide 91 videos in 5 groups 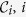 = 1, 2, 3, 4, 5. To compute the benignity-malignancy probability of a video in group 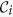 we train a classifier with the remaining four groups 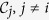. After repeating this process for each group, these probability values are treated as the output of the image based classifier. We evaluate the performance of each method in terms of area under the receiver operating characteristics (ROC) curve or AUC in short.

## Results

The clinical features of our clinical cohort are broadly consistent with the previously published cohorts (see supplementary information on *clinical characteristics of the cohort* for details).

### Classification using clinical information

**Figure 2:**
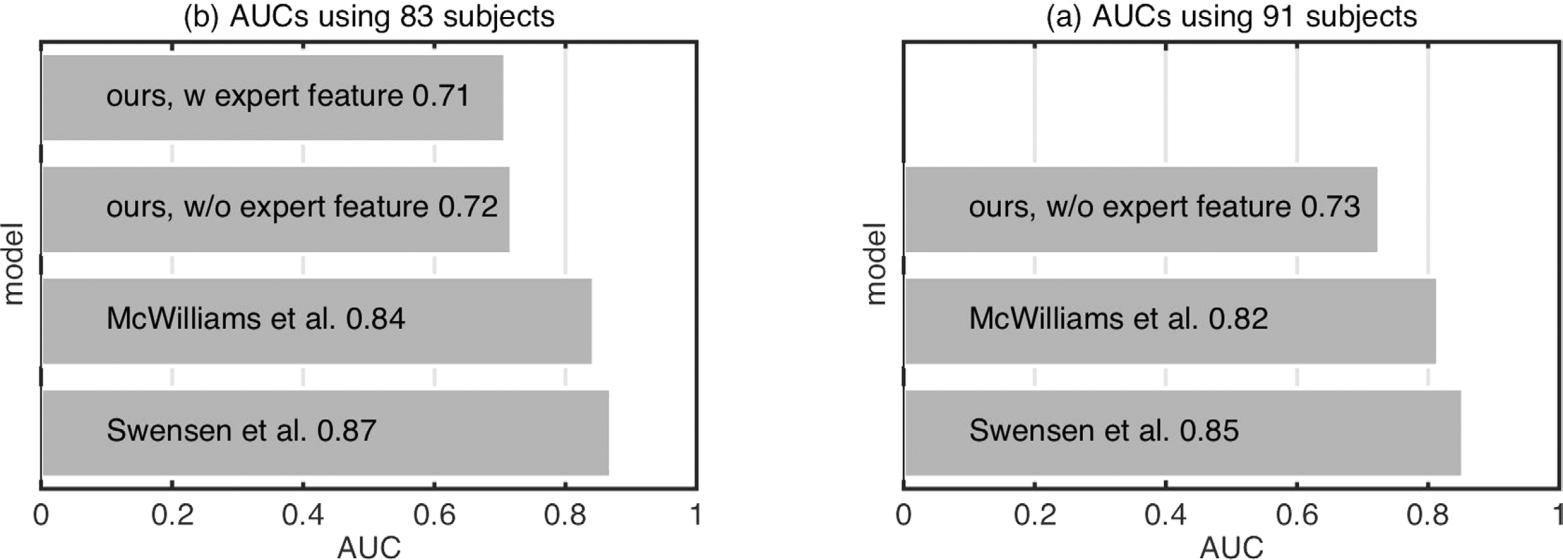
Comparison of existing models using clinical and demographic features^4^,^5^ versus models trained on our patient cohort either with or without using expert annotation as feature. AUCs are computed with either (**a**) 83 (for which expert annotation is available) or (**b**) 91 subjects (full cohort).

To assess the ability of an experienced FCFM operator in distinguishing the nodule type from FCFM texture, the on-target frames were reviewed by a blinded clinical expert. Each set of frames for a corresponding patient was annotated as benign or malignant, which was possible in 83 of the 91 videos. These annotations were then used as a feature along with the 12 features we have to predict malignancy. Figure 2a presents the AUC achieved by fitting the clinical information in the existing risk calculators^4^,^5^, and two linear logistic regression models trained by us with 12 features and 13 features respectively, where the 13-th feature is the annotations by the clinical expert. In the last two cases the output probabilities of the classifiers for each video is obtained by 5 fold cross-validation. We observe that both the models trained by us perform worse than the existing models. A possible reason for this might be that we use many fewer samples to learn the models compared to the previous studies. We also observe that the performance deteriorates slightly when we use expert annotations as feature, demonstrating the difficulty of the classification task since there is little visual difference between the two group of videos (see Fig. 1 in supplementary information for an illustration). Since we use all 91 subjects for evaluating our method in the following sections, we present the corresponding AUCs in Fig. 2b.

### Classification using imaging information

**Figure 3:**
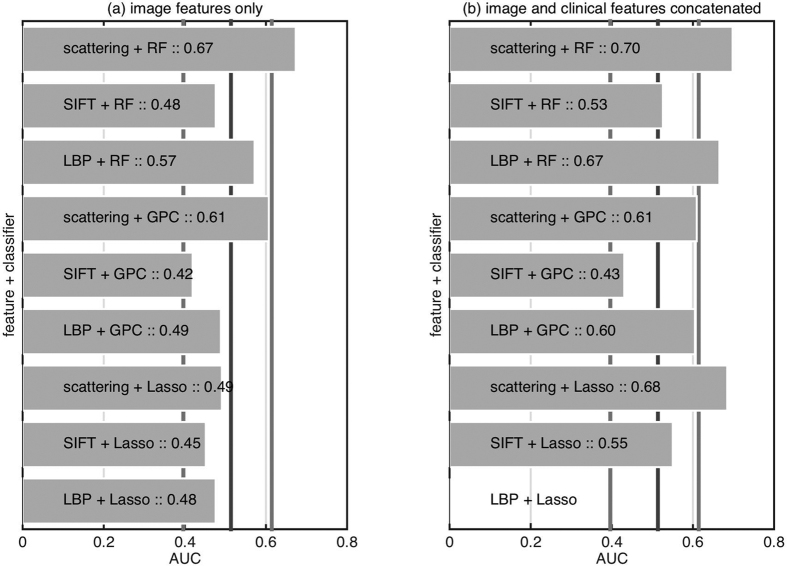
Comparison of different feature extraction and classification methods on predicting benign versus malignant nodules based on (left) only imaging features, and (right) imaging features concatenated with clinical features. The vertical lines denote the median, and (0.05, 0.95) quantiles of the empirical AUC distribution where the classifier output is random. LBP Lasso didn’t converge within given time for image and clinical features concatenated.

We assess the ability of FCFM imaging information alone in classifying subjects as benign or malignant. Figure 3a presents the performance of different feature extraction and classification methods. We also present the empirical distribution (0.05 and 0.95 quantiles) of AUC when the classifier output has been drawn randomly from a uniform distribution, where AUC ≈ 0.5 implies classification by random chance. We observe that almost all of the feature extraction and classification strategies perform within the random classifier confidence interval. However, scattering features with a nonlinear classifier do show an AUC above the confidence intervals. Figure 3b presents the performance of different feature extraction methods and different classifiers trained on image features and clinical features concatenated. Although the inclusion of clinical information improves the classification performance, it is still considerably below those of the risk calculators using clinical features alone.

### Classification using clinical and imaging information

**Figure 4:**
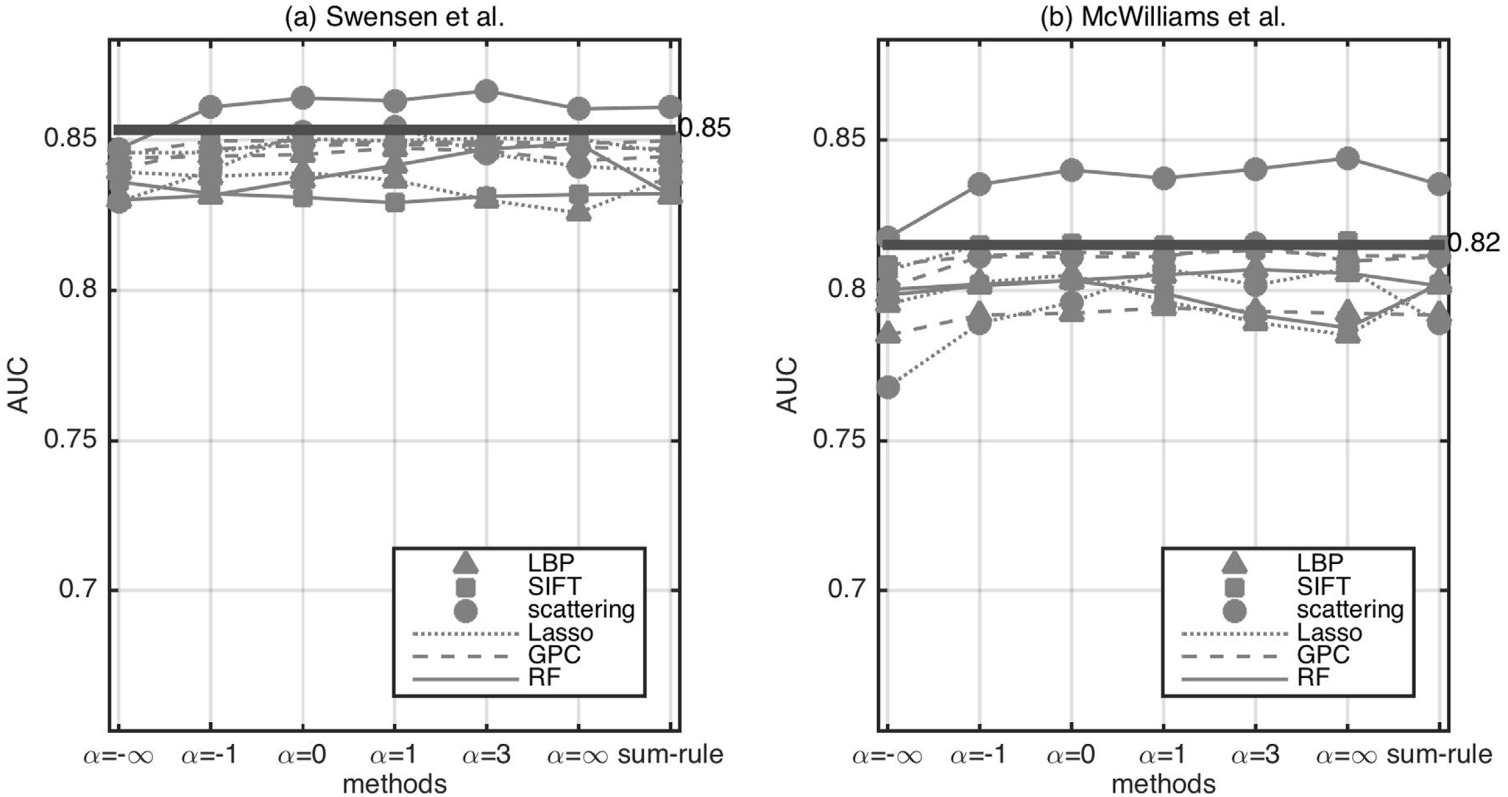
Comparison of different feature extraction, classification methods, and classifier combination strategies on predicting benign versus malignant nodule when combined with existing models based on clinical and demographic information (left) Swensen *et al*.^4^ (right) McWilliams *et al*.^5^. The horizontal line represents performance using only the existing model without imaging information.

**Figure 5:**
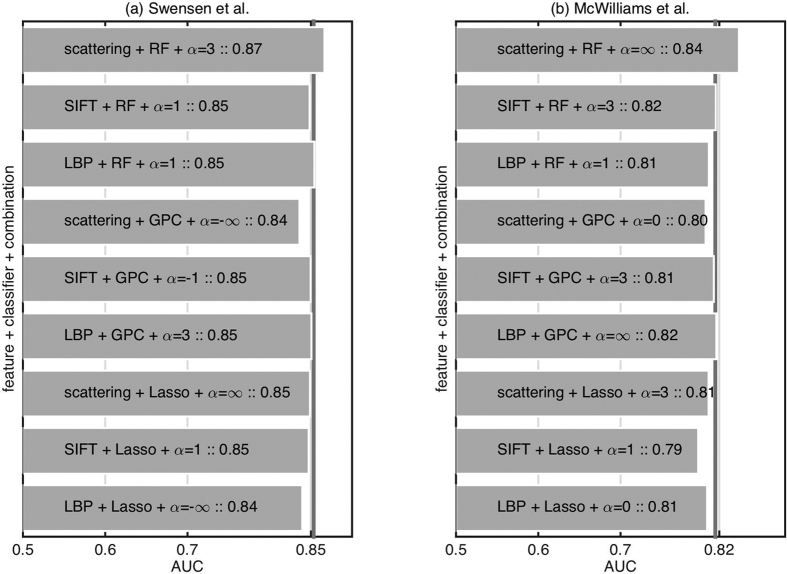
Comparison of different feature extraction and classification methods with best classifier combination strategy on predicting benign versus malignant nodule when combined with existing models based on clinical and demographic information (left) Swensen *et al*.^4^ (right) McWilliams *et al*.^5^. The vertical line represents performance using only the existing model without imaging information.

To assess the impact of the imaging information on the clinical risk calculator performance characteristics we combine the information from imaging data to existing risk calculators. For each feature extraction and classification strategy we choose the best classifier combination strategy (see Fig. 4). We choose this particular combination strategy without cross-validating over different strategies. This risks overfitting, but we avoided cross-validating over combination strategies so as to not reduce the training set further. However, as we conclude that even the best classifier combination strategy only results in marginal (not statistically significant) improvement, it is justified. Figure 5 presents the results of combining the imaging classifier outputs with existing models. We observe that only scattering features with RF shows promising result while the rest of the feature extraction and classifier combinations reduce the performance.

**Figure 6:**
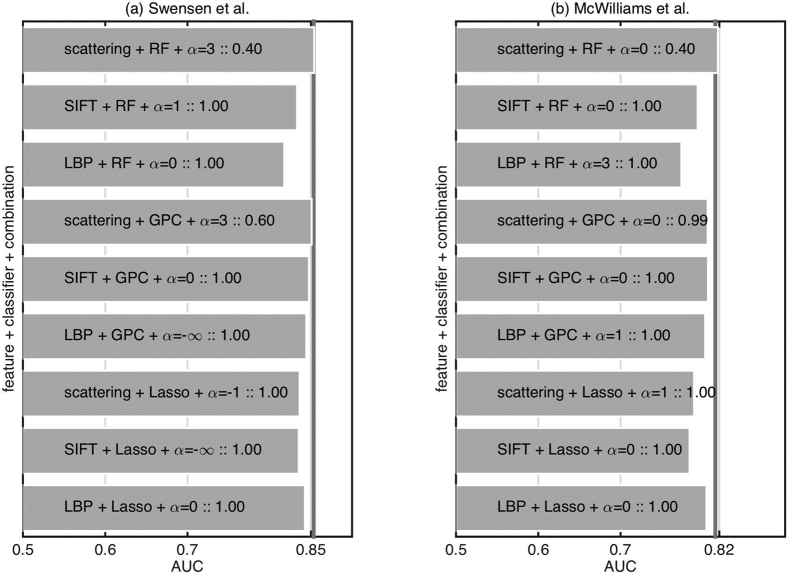
Comparison of different feature extraction and classification methods on predicting benign versus malignant nodule when combined with existing models based on clinical and demographic information (left) Swensen *et al*.^4^ (right) McWilliams *et al*.^5^. The bars are the median over 16 cross-validation splits, and the numbers are the p-value returned by a signtest to check if the median is above the vertical line. The vertical line represent performance using only the existing model without imaging information.

To test if the improvement over existing models is statistically significant or just random chance, we repeat the experiment with different cross-validation splits. Figure 6 presents the result of a signtest (implementation signtest available in Matlab) to see if the median AUC over different cross-validation splits is significantly greater than the existing models. We observe that only scattering features with the random forest classifier provide a promising result although not statistically significant (*p* = 0.40 in both cases).

## Discussion

Label-free FCFM allows imaging of autofluorescent lung parenchyma at cellular resolution, but we have demonstrated that there are no features seen on manual assessment or obtained through a automated feature extraction that improve the operator characteristics of nodule calculators over using clinical and CT radiological features alone in a large clinical cohort. We have used a non-biased automated approach to investigate the utility rather than rely solely upon human eye data extraction. Indeed, as autofluorescenec originates from elastin and collagen in the lung^9^, it is perhaps not surprising that the remodelled extracellular matrix present around both benign and malignant nodules is not distinguishable without molecular profiling. That said, a number of limitations to this work must be acknowledged. Firstly, this work formed part of a prospectively collected database, but is a retrospective analysis of the work, which carries inherent bias^24^. Secondly, there are the potential problems around imaging: although all imaging was performed by a single experienced expert operator, bias for the length of time imaging an abnormal area, and whether all on target frames were included in the analyses must be acknowledged as limitations to the work. This imaging modality also includes motion artefacts in the images, due to breathing and fibre movement, which may require removal before automated analysis. Thirdly, the interpretation of the data relies on sufficient contrast in the imaging data and a number of videos could not be interpreted manually due to the poor contrast. It is to be noted that the output of the FCFM imaging are intensity values of the autofluorescence, and the dynamic range of these values can be drastically different for different images, making it difficult to compare them. Furthermore, the imaging field of view is small compared to the nodule size, i.e., 600 microns compared to size of the nodule which may be up to 30 mm, and therefore, the clinician only has a partial view of the nodule. Whilst this field of view remains comparatively small and allows for high resolution imaging, this approach allows for imaging at multiple sites of the nodule penumbra in a minimally invasive way and has already been shown to demonstrate pathological features when used for proximal large airway tumours with topical dyes. Importantly, the penumbra of the tumour is the key area to image and sample as intratumoral necrosis is often present^25^. Fourthly, we decided to analyse images irrespective of smoking status, which can have impact on the accuracy of the image analysis^10^,^12^, as only 13 of the overall cohort were never smokers, and only 2 subjects that were never smokers developed malignant nodules. However, the approaches that we have pursued in this study could be further developed and applied in a cohort of never smokers who develop malignant nodules to interrogate if this approach of non-label autofluorescence endomicroscopy of nodules provides any additional features in this cohort. Finally, although the clinical data is complete, a number of cases had to be excluded due to missing/unreadable FCFM data. However, the patient cohort we analyze has a number of strengths: the clinical data is complete for the parameters assessed for risk calculation, the study demonstrates the feasibility of reaching pulmonary nodules by FCFM imaging, it contains a significant number of patients and it has been subject to robust feature extraction and analysis.

Given that the above approach does not show a significant advantage in using FCFM information, other approaches must be considered; the use of an internal control for each patient by acquiring FCFM images from a distinct bronchopulmonary segment either alone or in conjunction with generic contrast agents or the targeted use of molecularly targeted optical imaging agents may all increase performance^26^. The use of generic contrast agents for pulmonary FCFM images has previously been considered. The use of topical methylene blue^13^ have been used to demonstrate nuclear staining in pulmonary FCFM imaging but requires the 660nm Cellvizio system, where the autofluorescence would not be seen. Fluorophores with compatibility at 488 nm include acriflavine, which has been advocated by some groups^27^ but not others^28^ and cresyl violet, which has been demonstrated to provide contrast with a prototype fiber based confocal system for bronchial imaging^29^. Fluorescein, which has been extensively used and is well established with FCFM in gastrointestinal imaging to demonstrate cellular dysplasia and malignancy^30^, has met with limited success when administered intravenously for pulmonary FCFM imaging in humans^31^ but has been demonstrated for bronchial vasculature imaging in preclinical models^32^. Our group has also demonstrated the potential of targeted imaging with 488 nm compatible Smartprobes administered topically in whole large animal lung models and human lung tissue *ex vivo*^33^,^26^. The potential of fluorescein-based Smartprobe targeted imaging in lung cancer has also been demonstrated by targeting EGFR mutations in cell line xenograft mouse model^34^ and this approach has been demonstrated *in vivo* for urological conditions^35^ and oesophageal malignancy^36^. Therefore, the use of contrast agents or Smartprobes may allow the identification of features specifically associated with malignancy that are similar to histopathological features seen on biopsies.

In summary, in a clinically relevant cohort of patients with pulmonary nodules, this work demonstrates that label-free FCFM data does not improve operator characteristics of risk calculators to distinguish benign from malignant nodules. Therefore, future work in the detection of benign from malignant nodules will likely need to include fluorescence-based tracers to see cellular structures of the nodule, but ideally should include a targeted molecular labeling strategy. Pulmonary nodule label-free FCFM methods may likely only show utility to inform the clinician if the area is abnormal, and so label-free FCFM may be used to guide sites for biopsy to improve diagnostic yield.

## Additional Information

**How to cite this article**: Seth, S. *et al*. Assessing the utility of autofluorescene-based pulmonary optical endomicroscopy to predict the malignant potential of solitary pulmonary nodules in humans. *Sci. Rep*. **6**, 31372; doi: 10.1038/srep31372 (2016).

## Supplementary Material

Supplementary Information

## Figures and Tables

**Figure 1:**
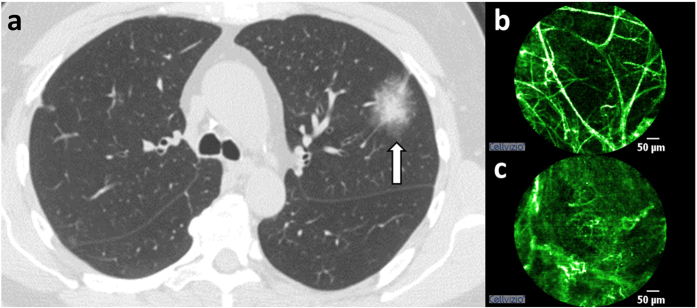
(**a**) CT scan demonstrating pulmonary nodule, (**b**) normal elastin structure, (**c**) abnormal elastin structure at nodule. Notice that abnormal structure may appear due to benign or malignant cause.
