# Supplementary material for: Assessing the utility of autofluorescence-based pulmonary optical endomicroscopy to predict the malignant potential of solitary pulmonary nodules in humans
Source: Sci Rep. 2016 Aug 23;6:31372. doi: 10.1038/srep31372 (PMC4993998; doi:10.1038/srep31372)
Supplement: Supplementary Information [file srep31372-s1.pdf]

Assessing the utility of autofluorescence-based pulmonary optical endomicroscopy to predict the malignant potential of solitary pulmonary nodules in humans

Sohan Seth[1,*,+], Ahsan R. Akram[2,+], Paul McCool[3], Jody Westerfeld[4], David Wilson[4], Stephen McLaughlin[3], Kevin Dhaliwal[2], and Christopher K. I. Williams[1]

[1]University of Edinburgh, School of Informatics, Edinburgh, EH8 9AB, UK
[2]University of Edinburgh, Queens Medical Research Institute, MRC Center for Inflammation Research, Pulmonary Molecular Imaging Group, Edinburgh, EH14 4TJ, UK
[3]Heriot-Watt, School of Engineering and Physical Sciences, Edinburgh, EH14 4AS, UK
[4]Community Health Network, Community South hospital, Indianapolis, IN 46227, USA
[*]sseth@inf.ed.ac.uk
[+]these authors contributed equally to this work

ABSTRACT

Supplementary information.

Risk calculators

One of the earliest works on predicting benign and malignant lung nodules was undertaken by Swensen et al.[1] The authors studied solitary pulmonary nodules (SNP) that are 4-30 mm in diameter, and suggested the following model for determining the probability of a nodule being benign or malignant.

$$\text{probability-of-malignant} = \frac{e^x}{1+e^x}, \text{ where}$$
$$x = (0.0391 \times \text{age}) + (0.7917 \times \text{smoker}) + (1.3388 \times \text{extra-thoracic-cancer}) + (0.1274 \times \text{nodule-size}) + (1.0407 \times \text{spiculation}) + (0.7838 \times \text{upper-lobe}) + 6.82724.$$

The authors built univariate classifiers for each feature, and then selected the most effective features (p-value < 0.001) for multivariate analysis to compute the probability of nodule being benign or malignant. The authors studied a total of 629 subjects with 65% benign, 23% malignant and 12% intermediate nodules. Benign cases can be granuloma (79%), hamartomas (7%) and others (14%). The model was trained on 419 samples and tested on 210 samples.

In recent years, the problem has been revisited by McWilliams et al.[2] The authors report similar analysis and findings as by,[1] e.g., in a univariate analysis, significant consistent predictors of lung cancer included the size, type (non-solid, part-solid, or solid), and location of the nodules, and the number of nodules that were detected. However, a crucial difference is that the size of the nodule was associated with lung cancer in a significant nonlinear relationship ($p < 0.001$ for nonlinearity). The nonlinear relationship was modeled with the use of multivariable fractional polynomials (see Figure 1 and Table 2 in the respective article for more details). Additionally, the authors train a parsimonius model (using only significant variables with p-values < 0.05) and a full model (with all variables) for two cases: one with spiculation as feature and other without spiculation as feature (the models have been presented in Table 2 of the respective article). We use the following model that is also available at McWilliams *et al.* calculator,

$$\text{probability-of-malignant} = \frac{e^x}{1+e^x}, \text{ where}$$
$$x = -6.7892 + (0.0287 \times (\text{ age } - 62)) + (0.6011 \times \text{ sex }) + (0.2961 \times \text{ family-history-of-lung-cancer }) + (0.2953 \times \text{ emphysema }) + (0.6581 \times \text{ upper-lobe }) - (0.0824 \times (\text{ number-of-nodules } - 4)) + (0.7729 \times \text{ spiculation }) - (5.3854 \times (\sqrt{10/\text{nodule-size}} - 1.58113883)) + (0.377 \times \text{ nodule-type }).$$

FCFM imaging

FCFM utilises a proximal laser scanning unit (LSU) emitting blue argon laser light at 488nm and transmits this down a flexible bundle, through the working channel of a bronchoscope, to the tissue being imaged. The most commonly used fiber for pulmonary imaging is 1.4mm in diameter and houses up to 30,000 individual fibers of varying diameter. It affords an axial resolution of 0-50 micrometers, a lateral resolution of 3.5 micrometers, a field of view of 600 micrometers and a scan rate of 12 frames per second.[3] The 488nm system can image distal human airways through autofluorescence of elastin[4,5] which represents up to 50% of distal lung connective tissue fibers. Contributions from endogenous flavins and collagen are lower when using a 488nm excitation source and occasionally, in smokers or ex-smokers, autofluorescent cells are also seen.[5]

FCFM image analysis

Image analysis for pulmonary FCFM imaging has been previously undertaken: Herbert et al.[6] analysed FCFM images of the distal lung in healthy volunteers, or patients with interstitial lung disease. A total of 133 images from never smokers, and 93 images from current or ex-smokers were included. Using local binary patterns (LBP) in a boosted cascade of classifiers they found that there was discrimination from healthy alveolar structure from pathological in 86.3% and 95.1% of non-smokers and smokers respectively.

Further work in the same group[7,8] demonstrated that classification between pathological and healthy in both smokers and non-smokers can be improved using image analysis techniques including application of random subwindows and extra-trees (with LBP) or support vector machine classifiers. Désir et al.[7] explore the task of separating normal lungs (nonsmoking or smoking healthy) from pathological lungs (diagnosed with lung disease from abnormal growth detected via CT scan) from FCFM images. The authors extract local binary patterns from the images and use a Extra-Trees classifier to solve this problem. However, instead of extracting LBP features exhaustively, the authors suggest taking random subwindows (random location, random width) to create a larger training set (40,000 subwindows for each class). The dataset explored by the authors comprises two separate groups of 133 nonsmokers and 93 smokers, and within each group some images are labelled healthy and some pathological. The authors report 92% accuracy using LBP features compared to 80% with images for nonsmoker group.

Désir et al.[8] explore the task of classifying healthy vs. pathological lungs from FCFM images, using a variety of image features (see Tables 1 and 2 in the respective article), and a support vector machine (SVM) classifier. The ground truth was obtained by "two medical experts" and "no correlation study between histology and FCFM features [was] intended in [the] work".[8] The suggested method relies on the structural differences in two groups of images: to elaborate, while the elastin structures in the healthy subjects are continuous lines and curves, it becomes disorganized in the pathological subset. Therefore, the authors concentrate on distinguishing this structural difference using texture related features, and they conclude that LBP features achieve the best result.

Finally, it has also demonstrated that with the application of methylene blue (a contrast agent) and using the Cellvizio system operating at 660nm wavelength for the diagnosis of bronchial lesions can report a 90% classification accuracy.[9] Rakotomamonjy et al.[9] explore the feasibility of lung cancer diagnosis from FCFM images. They demonstrate that *scattering features* are highly discriminative for this task (90% accuracy with spline wavelets). They also mention that LBP/LQP features improve classifier performance when concatenated with scattering features. The dataset they consider has 103 healthy images from 8 persons, and 70 images from 7 patients. The authors use a support vector machine (SVM) with Gaussian kernel as classifier. However, instead of a cross-validation type setup, the authors randomly split the dataset in training-testing where training set size can vary from 30% to 80% of the dataset. The training set is then split in training and validation to select hyperparameters for the classifier. On a technical note, the authors take the largest square in the circular image to extract scattering as well as LBP features, and the collection of images are downsampled to 128×128 for efficient computation of the scattering features.

The work by these groups in the extraction and classification of features from FCFM images in the lung are promising, demonstrate that computational analysis has a definite role in pulmonary FCFM imaging, and form the foundation for future work in this field. However, a number of significant differences remain with our work as we are assessing the difference between two pathological conditions, i.e. benign from malignant lesions, both of which cause abnormal alveolar fluorescence images. Furthermore, we are attempting to do this with label-free methods and their latter work uses topical dye administration. Additionally, a related aspect to be noted is that in existing studies one usually tackles smoking patients separately from nonsmoking patients due to the presence of distinguishing granular structure in the former group. However, we do not make such distinction, thus making the problem more difficult and yet more realistic. Finally, we extract information from a sequence of images for each subject rather than relying on a single image chosen by an expert. We have used a large dataset from 91 patients and include about 17,000 frames for analysis.

Representative frames

Figure 1 shows a representative on-target frame from example videos, computed over the longest on-target frame sequence. Representative frames were computed as medoids of the frames in 1401 dimensional feature space created by scattering features since scattering feature demonstrated favourable classification performance. It is to be observed that there is no striking visual difference between the two groups.

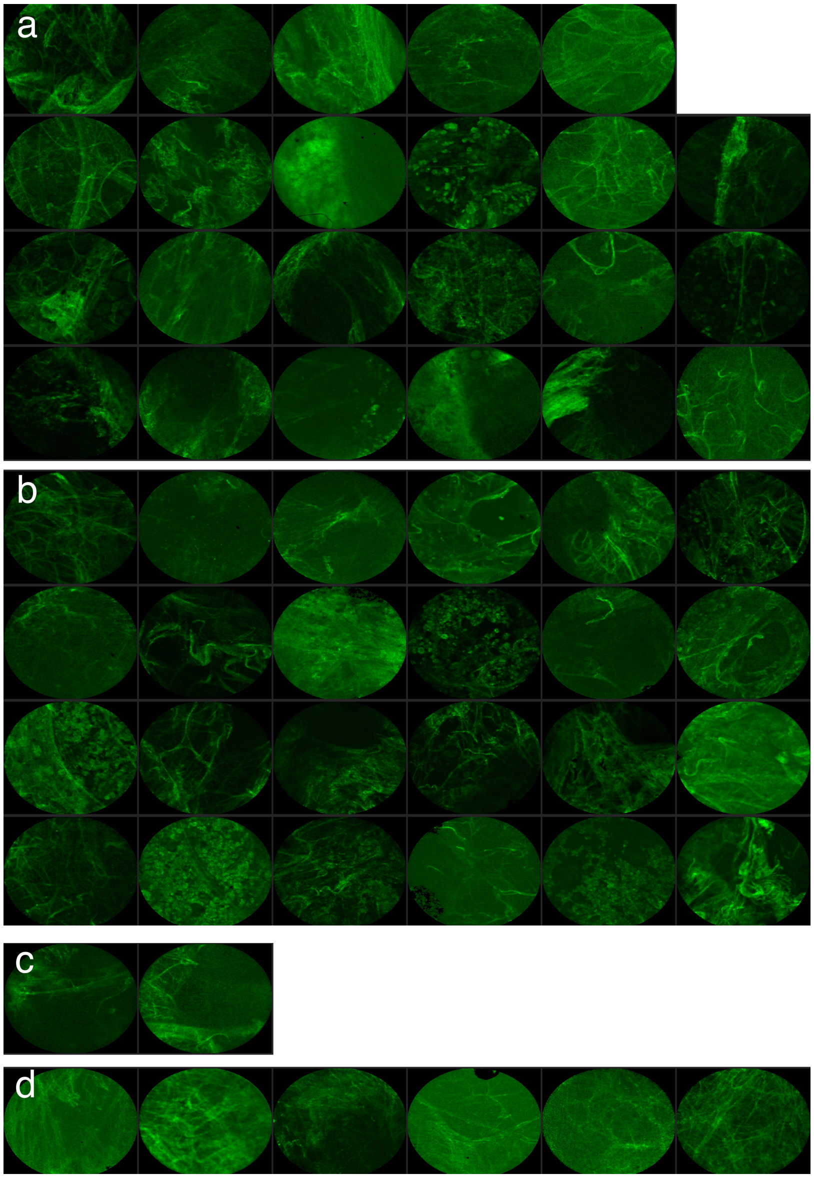

Figure 1. Examples of (a) malignant smokers or ex-smokers (b) benign smokers or ex-smokers (c) malignant never smokers and (d) benign never smokers frames. Each frame is the medoid of the longest continuous on-target frames of the respective video. For better visualization, we removed 'dark spots' in the image (region with negative intensity values over all on-target frames).

Combining classifiers

sum-rule[10] describe different rules for combining classifiers, and demonstrates that the *sum-rule* performs the best in practice potentially due to its robustness to estimation error. The standard rule for combining classifiers is

$$\arg\max_k \mathrm{p}(y_k|\mathbf{x}_1,\ldots,\mathbf{x}_p)$$

where $\mathbf{x}_i$'s are the input to the i-th classifier, $i = 1,\ldots,p$. Under the assumption of independence of $\mathbf{x}_i$s given class y_k one arrives at the product-rule

$$\arg\max_k \mathrm{p}(y_k)^{-(p-1)} \prod_{i=1}^{p} \mathrm{p}(y_k|\mathbf{x}_i).$$

The authors note that if one assumes that the posterior probabilities do not deviate from the prior much, i.e., $\mathrm{p}(y_k|\mathbf{x}_i) = P(y_k)(1+\delta_{ki})$, then one can approximate the product-rule by sum-rule as

$$\arg\max_k \left[(1-p)\mathrm{p}(y_k) + \sum_{i=1}^{p} \mathrm{p}(y_k|\mathbf{x}_i)\right].$$

α-integration Amari[11] suggests combining the output of two classifiers through generalized mean of the form $\mathrm{p}(y_k|\mathbf{x}_1,\ldots,\mathbf{x}_p) = cf^{-1}(\sum_i f(\mathrm{p}(y_k|\mathbf{x}_i)))$ where f is a suitable monotone transformation $f(u) = u^{\frac{1-\alpha}{2}}$ if $\alpha \neq 1$ and $\log u$ if $\alpha = 1$, and c is a normalizing constant. By changing α one gets a family of generalized means with the arithmetic and geometric means as special cases, e.g.,

$$\alpha = -\infty :: m_{-\infty}(a,b) = \max\{a,b\}$$
$$\alpha = -1 :: m_{-1}(a,b) = \frac{1}{2}(a+b)$$
$$\alpha = 0 :: m_0(a,b) = \frac{1}{4}(\sqrt{a}+\sqrt{b})^2$$
$$\alpha = 1 :: m_1(a,b) = \sqrt{ab}$$
$$\alpha = 3 :: m_3(a,b) = \frac{2}{\frac{1}{a}+\frac{1}{b}}$$
$$\alpha = \infty :: m_\infty(a,b) = \min\{a,b\}.$$

where a and b are two probability values. It is worth noting that $m_\alpha(a,b) \geq m_{\alpha'}(a,b)$ if $\alpha \geq \alpha'$, and also, as α is increased more importance is given to the lower of the two values (a and b). Using α integration the combined probability distribution is given by $\mathrm{p}(y_k|\mathbf{x}_1,\mathbf{x}_2) = cm_\alpha(\mathrm{p}(y_k|\mathbf{x}_1),\mathrm{p}(y_k|\mathbf{x}_2))$ where $\mathbf{x}_1$ and $\mathbf{x}_2$ are two clinical and imaging features respectively.[11] shows that α-integration is meaningful in the sense that it minimizes the α-divergence between the combined distribution and the candidate distributions (see Theorem 2 in[11]).

Clinical characteristics of the cohort

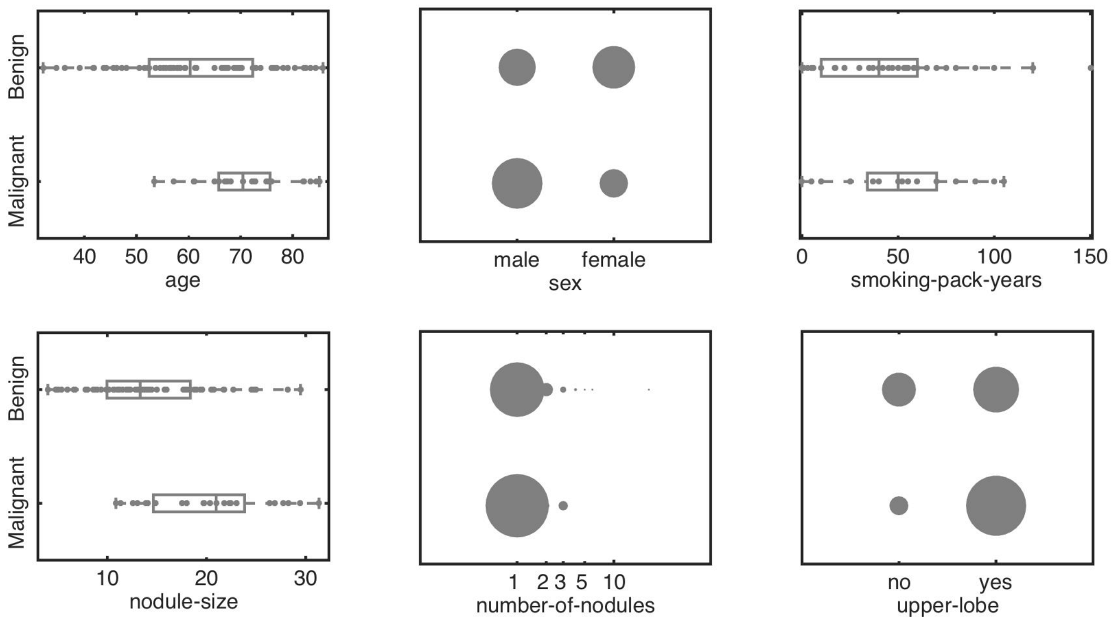

Figure 2. Illustration of clinical characteristics of the patient cohort. For age, smoking pack years, number of nodules, and nodule size, we report the respective quartiles for comparison. We present sex, number of nodules, and upper lobe as bubble plots where the size of the bubble represents percent of times the variable takes the value.

The clinical cohort consisted of 91 patients with complete clinical, radiological and FCFM data. Figure 2 demonstrates the clinical characteristics of the cohort which are broadly consistent with previous published cohorts.[1,2,12–14] This dataset confirms that malignant nodules are found in older patients (AGE), have a larger axial diameter (NODULE-SIZE), have an upper lobe predominance (UPPER-LOBE). This cohort has an incidence of malignancy of 28% and although the literature reports variable incidences of malignancies depending on the population studied, a prevalence of 20-30% is reported in a systematic review.[14] Furthermore, for the Mayo clinic model[1] this data demonstrated an area under the curve (AUC) of 0.87, which is higher than the reported literature of external validation of the model. The Mayo clinic model has been modified to incorporate a PET-CT result[13] but found a baseline (pre-PET) AUC of 0.79, which is similar to the validation cohorts of[15] with AUC of 0.80 and Isbell et al[16] of 0.78 for the Mayo clinic model. However, in all these cohorts the incidence of malignancy was higher with 57%, 44% and 73% respectively, whereas the initial cohort from Mayo model[1] reported an incidence of 23% and the AUC was 0.83. The Brock model[2] has not been externally validated and taken together, these results demonstrate this performs worse than in the initial study, which included a validation cohort and the AUC was reported as 0.96.[2] Taken together, the results demonstrate that this cohort is broadly consistent with the published literature on pulmonary nodule cohorts and the data reflects the real-life management of patients with pulmonary nodules.

References

1. Swensen, S. J., Silverstein, M. D., Ilstrup, D. M., Schleck, C. D. & Edell, E. S. The probability of malignancy in solitary pulmonary nodules. *Arch Int Med* **157**, 849–855 (1997).
2. McWilliams, A., Tammemagi, M. C. & Lam, S. Probability of cancer in pulmonary nodules detected on first screening. *N Engl J Med* **369**, 910–919 (2013).
3. Thiberville, L. *et al.* Confocal fluorescence endomicroscopy of the human airways. *Proc Am Thorac Soc* **6**, 444–9 (2009a).
4. Thiberville, L. *et al.* In vivo imaging of the bronchial wall microstructure using fibered confocal fluorescence microscopy. *Am J Respir Crit Care Med* **175**, 22–31 (2007).
5. Thiberville, L. *et al.* Human in vivo fluorescence microimaging of the alveolar ducts and sacs during bronchoscopy. *Eur Respir J* **33**, 974–985 (2009).
6. Hébert, D., Désir, C., Petitjean, C., Heutte, L. & Thiberville, L. Detection of pathological condition in distal lung images. In *Proc. ISBI*, 1603–1606 (2012).
7. Désir, C., Petitjean, C., Heutte, L., Salaün, M. & Thiberville, L. Classification of endomicroscopic images of the lung based on random subwindows and extra-trees. *IEEE T Bio-Med Eng* **59**, 2677–2683 (2012).
8. Désir, C., Petitjean, C., Heutte, L., Thiberville, L. & Salaün, M. An SVM-based distal lung image classification using texture descriptors. *Comput Med Imag Grap* **36**, 264–270 (2012).
9. Rakotomamonjy, A., Petitjean, C., Salaün, M. & Thiberville, L. Scattering features for lung cancer detection in fibered confocal fluorescence microscopy images. *Artif Intell Med* **61**, 105 – 118 (2014).
10. Kittler, J., Hatef, M., Duin, R. P. W. & Matas, J. On combining classifiers. *IEEE T Pattern Anal* **20**, 226–239 (1998).
11. Amari, S.-I. Integration of stochastic models by minimizing α-divergence. *Neural Comput* **19**, 2780–2796 (2007).
12. Gould, M. K. *et al.* Evaluation of patients with pulmonary nodules: when is it lung cancer?: ACCP evidence-based clinical practice guidelines (2nd edition). *Chest* **132**, 108S–130S (2007).
13. Herder, G. J. *et al.* The performance of(18)f-fluorodeoxyglucose positron emission tomography in small solitary pulmonary nodules. *Eur J Nucl Med Mol* **31**, 1231–6 (2004).
14. Wahidi, M. M., Govert, J. A., Goudar, R. K., Gould, M. K. & McCrory, D. C. Evidence for the treatment of patients with pulmonary nodules: when is it lung cancer?: ACCP evidence-based clinical practice guidelines (2nd edition). *Chest* **132**, 94S–107S (2007).
15. Schultz, E. M. *et al.* Validation of two models to estimate the probability of malignancy in patients with solitary pulmonary nodules. *Thorax* **63**, 335–41 (2008).
16. Isbell, J. M. *et al.* Existing general population models inaccurately predict lung cancer risk in patients referred for surgical evaluation. *Ann. Thorac. Surg.* **91**, 227–33 (2011).
